# Diet and inflammatory bowel disease: The Asian Working Group guidelines

**DOI:** 10.1007/s12664-019-00976-1

**Published:** 2019-07-27

**Authors:** Ajit Sood, Vineet Ahuja, Saurabh Kedia, Vandana Midha, Ramit Mahajan, Varun Mehta, Ritu Sudhakar, Arshdeep Singh, Ajay Kumar, Amarender Singh Puri, Bailuru Vishwanath Tantry, Babu Ram Thapa, Bhabhadev Goswami, Banchha Nidhi Behera, Byong Duk Ye, Deepak Bansal, Devendra Desai, Ganesh Pai, Ghulam Nabi Yattoo, Govind Makharia, Hasitha Srimal Wijewantha, Jayanthi Venkataraman, K. T. Shenoy, Manisha Dwivedi, Manoj Kumar Sahu, Meenakshi Bajaj, Murdani Abdullah, Namrata Singh, Neelanjana Singh, Philip Abraham, Rajiv Khosla, Rakesh Tandon, S. P. Misra, Sandeep Nijhawan, Saroj Kant Sinha, Sawan Bopana, Sheela Krishnaswamy, Shilpa Joshi, Shivram Prasad Singh, Shobna Bhatia, Sudhir Gupta, Sumit Bhatia, Uday Chand Ghoshal

**Affiliations:** 1 Department of Gastroenterology, Dayanand Medical College and Hospital, Ludhiana, 141 001 India; 2 Department of Gastroenterology, All India Institute of Medical Sciences, New Delhi, 110 023 India; 3 Department of Internal Medicine, Dayanand Medical College and Hospital, Ludhiana, 141 001 India; 4 Department of Dietetics, Dayanand Medical College and Hospital, Ludhiana, 141 001 India; 5 BLK Super Speciality Hospital, New Delhi, 110 005 India; 6 Department of Gastroenterology, GB Pant Hospital, New Delhi, 110 002 India; 7 Department of Gastroenterology, Kasturba Medical College, Mangalore, 575 001 India; 8 Department of Gastroenterology Postgraduate Institute of Medical Education and Research, Chandigarh, 160 012 India; 9 Department of Gastroenterology, Gauhati Medical College, Guwahati, 781 032 India; 10 Department of Dietetics, Postgraduate Institute of Medical Education and Research, Chandigarh, 160 012 India; 11 Department of Gastroenterology, Asan Medical Center, Seoul, South Korea; 12 Consultant Gastroenterology, Bathinda, 151 001 India; 13 P. D. Hinduja Hospital and Medical Research Centre, Mumbai, 400 016 India; 14 Department of Gastroenterology, Kasturba Medical College, Manipal, 576 104 India; 15 Sher-I-Kashmir Institute of Medical Sciences, Srinagar, 190 011 India; 16 Colombo North Teaching Hospital, Ragama, Sri Lanka; 17 Dr Kamakshi Memorial Hospital, Chennai, 600 100 India; 18 Department of Gastroenterology, Sree Gokulum Medical College and Research Foundation, Trivandrum, 695 011 India; 19 Department of Gastroenterology, Moti Lal Nehru Medical College, Allahabad, 211 001 India; 20 Department of Gastroenterology, IMS and Sum Hospital, Bhubaneswar, 756 001 India; 21 Dietician, Kilpauk, Chennai, 600 010 India; 22 Department of Internal Medicine, Faculty of Medicine Universitas Indonesia, Dr. Cipto Mangunkusumo General Hospital, Jakarta, Indonesia; 23 Department of Gastroenterology and Human Nutrition, All India Institute of Medical Sciences, New Delhi, 110 023 India; 24 Dietician, Pushpawati Singhania Research Institute, New Delhi, 110 001 India; 25 P D Hinduja Hospital and Medical Research Centre, Veer Savarkar Marg, Cadel Road, Mahim, Mumbai, 400 016 India; 26 Max Super Speciality Hospital, Saket, New Delhi, 110 017 India; 27 Pushpawati Singhania Research Institute, New Delhi, 110 001 India; 28 Department of Gastroenterology, Moti Lal Nehru Medical College, Allahabad, 211 001 India; 29 Department of Gastroenterology, SMS Medical College, Jaipur, 302 004 India; 30 Department of Gastroenterology, Postgraduate Institute of Medical Education and Research, Chandigarh, 160 012 India; 31 Fortis Hospital, Vasant Kunj, New Delhi, 110 070 India; 32 Dietician, Bengaluru, 560 001 India; 33 Dietician, Mumbai Diet and Health Centre, Mumbai, 400 001 India; 34 Department of Gastroenterology, Sriram Chandra Bhanj Medical College and Hospital, Cuttack, 753 001 India; 35 Department of Gastroenterology, King Edward Memorial Hospital, Mumbai, 400 012 India; 36 Shubham Gastroenterology Centre, Nagpur, 440 001 India; 37 Consultant Gastroenterology, Medanta The Medicity, Gurgaon, 122 001 India; 38 Department of Gastroenterology, Sanjay Gandhi Postgraduate Institute of Medical Sciences, Lucknow, 226 014 India

**Keywords:** Diet, Dietary practices, Guidelines, Inflammatory bowel disease

## Abstract

**Introduction:**

These Asian Working Group guidelines on diet in inflammatory bowel disease (IBD) present a multidisciplinary focus on clinical nutrition in IBD in Asian countries.

**Methodology:**

The guidelines are based on evidence from existing published literature; however, if objective data were lacking or inconclusive, expert opinion was considered. The conclusions and 38 recommendations have been subject to full peer review and a Delphi process in which uniformly positive responses (agree or strongly agree) were required.

**Results:**

Diet has an important role in IBD pathogenesis, and an increase in the incidence of IBD in Asian countries has paralleled changes in the dietary patterns. The present consensus endeavors to address the following topics in relation to IBD: (i) role of diet in the pathogenesis; (ii) diet as a therapy; (iii) malnutrition and nutritional assessment of the patients; (iv) dietary recommendations; (v) nutritional rehabilitation; and (vi) nutrition in special situations like surgery, pregnancy, and lactation.

**Conclusions:**

Available objective data to guide nutritional support and primary nutritional therapy in IBD are presented as 38 recommendations.

## Introduction

Inflammatory bowel disease (IBD) is a chronic relapsing–remitting immune disorder of unknown etiology that afflicts millions of individuals around the world with debilitating symptoms. The etiopathogenesis of IBD is complex and involves an interaction of genetic, environmental, and microbial factors and immunological responses [1]. An increasing incidence of IBD in populations where it was seen uncommonly in the past suggests an important role for environmental factors in its development [2–4]. Among the various environmental factors, diet seems to play an important role. The increasing incidence of IBD in developing countries parallels the westernization of diet, which includes an increased intake of food rich in fat and protein and a lesser intake of fiber and fruits. No single item is likely to be responsible; hence, it is important to study the dietary practices, which keep changing with time and are apparently different in Asian and the Western countries.

The Asian Working Group on diet in IBD thus met with the intentions to identify the role of diet in the pathogenesis and therapy of patients with IBD, assess the nutritional status of the patients, and to lay down recommendations on diet in Asian patients with IBD, which are summarized in this article.

## Methodology

### Sources and search

A comprehensive literature search was carried out for relevant articles published until 2018 on the role of diet in IBD. Search results obtained from PubMed, Medline, CENTRAL (Cochrane Central Register of Controlled Trials), InMED, Scopus, Embase, and Google were refined to include the most appropriate English literature. All the guidelines, original articles published on diet in IBD (both Asian and international), systematic reviews, meta-analyses, and review articles were included. Medical Subject Headings (MeSH terms) used were ulcerative colitis, Crohn’s colitis, inflammatory bowel disease, IBD, bowel diseases, Crohn disease, Crohn’s disease, Crohn’s enteritis, diet, enteral feeding, enteral nutrition, and malnutrition.

### Consensus process

A modified Delphi process was used to arrive at the consensus [5]. Based on the literature search, statements were proposed and these were circulated among eminent gastroenterologists who are key opinion leaders (KOLs) of their field and region. Prior to sharing the proposed statements for voting, consent was sought from each prospective participant. Six areas pertaining to the role of diet in IBD were identified viz. pathogenesis, therapy, malnutrition and nutritional assessment in a patient with IBD, dietary recommendations, and nutritional rehabilitation and special situations: surgery, pregnancy, and lactation. The questionnaire had one section for each of the areas and the proposed statements were designed according to issues of clinical importance in that area. Each statement had five voting options: accept completely, accept with some reservation, accept with major reservation, reject with reservation, and reject completely, with an option to make individual comments.

**Fig. 1:**
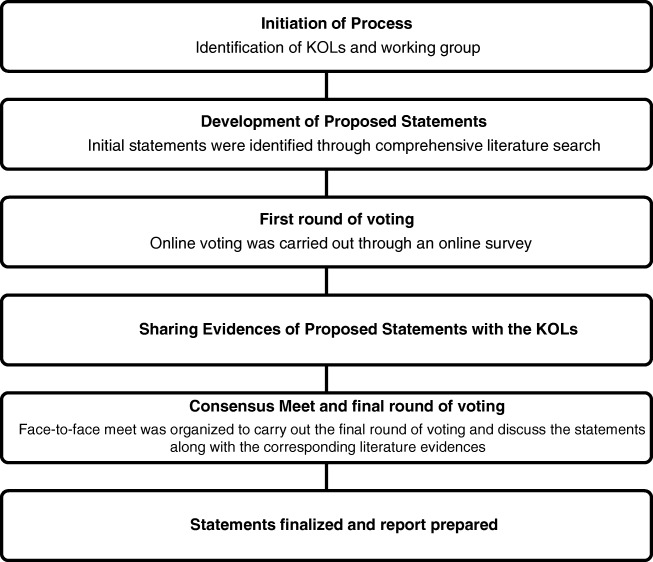
A modified Delphi process (Turoff and Linstone [5]). *KOL* key opinion leaders

First round of voting was held through an anonymous online survey. After the first round of voting, the available pieces of evidence for proposed statements in the questionnaire were shared with 30 KOLs. A face-to-face meeting was then held on 25 February 2018 at Dayanand Medical College and Hospital, Ludhiana, India, where evidences from the literature were presented for each proposed statement. Based on discussions among the gastroenterologists and the dieticians, a second round of voting (live anonymous vote using voting pads) was carried out during the meeting and consensus was achieved for each statement. Consensus on a statement was considered to be achieved when 80% or more voting members chose to “accept completely” or “accept with some reservation”. A statement was considered to be refuted when 80% or more of the voting members indicated “reject completely” or “reject with some reservation.” When no consensus was reached on a particular statement, it was modified and a second vote sought. If the second vote also remained inconclusive, the statement was deleted. The recommendations which received more than 80% agreements were finally accepted. The method used for development of the consensus, based on the modified Delphi process, is shown in Fig. 1.

### Grading of evidence

Grading of the level of evidence and grade of recommendation was then done by the participants using the scheme used by Indian Society of Gastroenterology Task Force consensus on ulcerative colitis [6], which was a modified version of the scheme suggested by the Canadian Task Force on Periodic Health Examination [7]. As shown in Table 1, the level of evidence is divided into I, II-1, II-2, II-3, and III; and grade of recommendation is classified as A, B, C, D, and E.

**Table 1:** Grade of recommendation and level of evidence

Quality of evidence		Strength of recommendation		Voting recommendation	
Grade	Description	Grade	Description	Option	Description
I	Evidence obtained from at least one randomized controlled trial	A	There is good evidence to support the statement	A	Accept completely
II-1	Evidence from well-controlled trials without randomization	B	There is fair evidence to support the statement	B	Accept with some reservation
II-2	Evidence from well-designed cohort or case–control study	C	There is poor evidence to support the statement	C	Accept with major reservation
II-3	Evidence from comparison between time or place with or without intervention	D	There is fair evidence to refute the statement	D	Reject with reservation
III	Opinion of experienced authorities and expert committees	E	There is good evidence to refute the statement	E	Reject completely

ISGTF Refer to the Indian Society of Gastroenterology Task Force Consensus on ulcerative colitis methodology (Ramakrishna et al. [6]), which was a modified version of the scheme suggested by the Canadian Task Force on the Periodic Health Examination (“The periodic health examination. Canadian Task Force on the Periodic Health Examination,” 1979 [7])

## The Asian Working Group guidelines regarding diet in inflammatory bowel disease

The statements recommended during the final meet are provided and include the level of supporting evidence, grade of recommendation, and voting results. This is followed by a discussion of the supporting evidence. A summary of the recommended statements is provided in Table 2.

**Table 2:** Summary of consensus recommendations for the medical management of inflammatory bowel disease

S no.	Statements
Role of diet in the pathogenesis of inflammatory bowel disease	
1)	Diet has an important role in the pathogenesis of inflammatory bowel disease (IBD), both ulcerative colitis (UC) and Crohn’s disease (CD). *Grade of recommendation: A, level of evidence: II-2*
2)	Epidemiological studies indicate that adoption of Western diet (low in fruits and vegetables, rich in fats, ω-6 fatty acids, red meat, and processed foods) contributes to the increasing incidence of IBD in developing countries. *Grade of recommendation: A, level of evidence: II-2*
3)	Dietary constituents like maltodextrins and emulsifiers may have a role in the development of IBD. *Grade of recommendation: B, level of evidence: II-3*
4)	Vitamin D may have a protective role in the natural history of IBD. *Grade of recommendation: B, level of evidence: II-2*
5)	Breastfeeding may have a protective role in the development of IBD. *Grade of recommendation: A, level of evidence: II-2*
Diet as a therapy for IBD	
6)	Exclusive enteral nutrition (EEN) is as effective as steroids in inducing remission in children with luminal Crohn’s disease. *Grade of recommendation: A, level of evidence: I*
7)	EEN is effective in adult CD but is inferior to corticosteroids for inducing remission. *Grade of recommendation: B, level of evidence: I*
8)	There is no difference between elemental and polymeric formulae in terms of efficacy. *Grade of recommendation: A, level of evidence: I*
9)	Partial enteral nutrition has been documented to be useful for maintenance of remission in luminal CD along with pharmacotherapy. *Grade of recommendation: A, level of evidence: I*
10)	More evidence is required, before elimination diets such as specific carbohydrate diet (SCD), Crohn’s disease exclusion diet (CDED), semi-vegetarian diet, anti-IBD diet, or low FODMAP (fermentable oligosaccharides, disaccharides, monosaccharides, and polyols) can be recommended as a therapy for CD. *Grade of recommendation: A, level of evidence: II-2*
11)	There is no specific role for exclusive (EEN) or partial enteral nutrition (PEN) for induction or maintenance of remission in patients with UC. *Grade of recommendation: B, level of evidence: I*
Malnutrition and nutritional assessment in a patient with IBD	
12)	Patients with IBD are at a higher risk of malnutrition hence all patients with IBD should be screened for malnutrition at presentation. *Grade of recommendation: B, level of evidence: II-2*
13)	The prevalence of malnutrition in a patient with IBD depends upon disease subtype, severity, extent, and duration. *Grade of recommendation: B, level of evidence: II-2*
14)	Body mass index alone is not sufficient for nutritional assessment of a patient with IBD. *Grade of recommendation: B, level of evidence: II-2*
15)	Dieticians/nutritionists should be involved in nutrition care of patients with IBD. *Grade of recommendation: B, level of evidence: I*
Dietary recommendations in IBD	
16)	For admitted patients with acute severe ulcerative colitis, adequate oral caloric intake is preferred to bowel rest. *Grade of recommendation: A, level of evidence: II-1*
17)	After stabilization of acute severe ulcerative colitis, a standard diet should be gradually introduced and oral nutritional supplements should not be a routine. *Grade of recommendation: B, level of evidence: III*
18)	For active inflammatory Crohn’s disease, oral diet with high protein is preferred to total parenteral nutrition. *Grade of recommendation: A, level of evidence: I*
19)	Once in remission, there is no need for diet modification or restriction and the patients can continue a normal diet as other family members. *Grade of recommendation: B, level of evidence: I*
20)	No dietary item in particular is established to cause relapse of disease activity in a patient in remission. *Grade of recommendation: B, level of evidence: II-1*
21)	Milk should not be routinely restricted in all patients with IBD unless patient has severe hypolactasia. *Grade of recommendation: A, level of evidence: I*
22)	A gluten-free diet (GFD) is not of a proven value in patients with IBD. *Grade of recommendation: B, level of evidence: II-3*
23)	A low FODMAP diet may help in alleviating irritable bowel syndrome (IBS)-like symptoms associated with IBD. *Grade of recommendation: A, level of evidence: I*
Nutritional rehabilitation in IBD patients	
24)	Patients with IBD should receive adequate calories, proteins and fats in their diet. The calorie and protein requirement of a patient with IBD in remission is similar to that of a healthy individual. However, the protein requirement is increased in a patient with active disease. *Grade of recommendation: B, level of evidence: III*
25)	Patients with IBD who have anemia should be evaluated appropriately for the cause of anemia and adequately treated. *Grade of recommendation: B, level of evidence: III*
26)	Proactive screening for osteopenia and its treatment should be done as per guidelines. *Grade of recommendation: A, level of evidence: III*
27)	Patients should be screened for micronutrient deficiency including calcium, phosphate, magnesium, iron, folic acid, and vitamin B_12_ in an appropriate clinical context. *Grade of recommendation: B, level of evidence: III*
28)	Except for patients with stricturing CD, there is no evidence for recommending either a low or a high fiber diet for patients with IBD. *Grade of recommendation: A, level of evidence: I*
29)	Patients with IBD should refrain from alcohol consumption as it may worsen the symptoms of disease. *Grade of recommendation: B, level of evidence: II-2*
30)	Patients of IBD should be encouraged to refrain from smoking. *Grade of recommendation: A, level of evidence: II-2*
31)	There is no scientific evidence to recommend probiotics as a food supplement. *Grade of recommendation: A, level of evidence: I*
32)	The nutritional status of patients with IBD should be optimized prior to elective surgery for a better outcome. *Grade of recommendation: B, level of evidence: III*
33)	If the nutritional goals cannot be met with an oral diet alone, oral nutritional supplements (ONS) or enteral nutrition should be initiated prior to surgery/perioperative phase. *Grade of recommendation: B, level of evidence: III*
34)	In elective surgery, the ERAS (early/enhanced recovery after surgery) protocol should be followed in the perioperative period. *Grade of recommendation: C, level of evidence: III*
Special situations: surgery, ostomies, pregnancy, lactation	
35)	Oral diet/EN should be started as soon as the patient can tolerate in the postoperative period. *Grade of recommendation: A, level of evidence: I*
36)	In the postoperative period, if oral diet cannot be resumed within 7 days, then enteral/parenteral nutrition should be initiated. *Grade of recommendation: A, level of evidence: I*
37)	In CD patients with a fistula, the type of diet depends upon the location of fistula–oral feeds for distal (low ileal or colonic) and low output fistula, and partial or exclusive parenteral nutrition for proximal and high output fistula. *Grade of recommendation: B, level of evidence: II-3*
38)	IBD patients with pregnancy should be specifically evaluated for iron and folate deficiency and replacement done accordingly. Recommended Dietary Allowances (RDA) for pregnancy and lactation should be followed. *Grade of recommendation: B, level of evidence: III*

### Role of diet in the pathogenesis of IBD

1. Diet has an important role in the pathogenesis of inflammatory bowel disease (IBD), both ulcerative colitis (UC) and Crohn’s disease (CD).

#### Grade of recommendation: A, level of evidence: II-2, voting: 92.1% agreement (A: 50%, B: 42.1%, C: 7.9%)

A dramatic increase in the incidence of IBD during the past 50 years is possibly related to environmental factors such as food and dietary habits that appear to be critical modulators of gut microbiota, which is one of the key elements in initiation of inflammation in IBD. Multiple dietary components may cause gut dysbiosis, both by altering the composition and microbial function. These apart, other indirect effects related to dietary factors that initiate inflammation and trigger immune response are damage to the mucus layer of the gut by metabolites of microbes [8].

Most studies evaluating the effect of diet on the risk of IBD have been retrospective case–control studies. The first systematic review published by Hou et al. in 2011 included 2609 IBD patients (1269 with CD, 1340 UC) and over 4000 controls from 19 studies [3]. Pre-illness intake of nutrients and risk of subsequent diagnosis of IBD were analyzed. A high dietary intake of total fats, polyunsaturated fatty acids (PUFAs), ω-6 fatty acids, and meat was associated with an increased risk of both CD and UC. Fiber- and fruit-rich diet was associated with low risk for CD, while vegetable-predominant diet was associated with decreased risk for UC [9]. Important insights have also emerged on role of diet in pathogenesis of IBD from other prospective studies in IBD, such as the European Prospective Investigation into Cancer and Nutrition (EPIC) cohort and the Nurses Health studies I and II cohorts [10, 11].

2. Epidemiological studies indicate that adoption of Western diet (low in fruits and vegetables, rich in fats, ω-6 fatty acids, red meat, and processed foods) contributes to the increasing incidence of inflammatory bowel disease in developing countries.

#### Grade of recommendation: A, level of evidence: II-2, voting: 82.4% agreement (A: 29.4%, B: 53%, C: 5.9%)

In a large prospective cohort of 170,776 female registered nurses (Nurses’ Health Study) followed over 26 years, it was observed that high intake of fiber, with fruits being the predominant source, was associated with a significant reduction in risk of CD but not UC [9]. This association was to a lesser degree from fiber of vegetables, and no association was identified from other sources of fiber such as cereals, whole grains, or legumes. A recent meta-analysis of 14 case–control studies concluded that consumption of vegetables was associated with decreased risk of UC alone, while higher consumption of fruits was associated with decreased risk of both UC and CD [12]. On subgroup analysis, the negative association of risk of CD with vegetables was observed from Europe but not from Asia. Another prospective study of women enrolled in the Nurses’ Health Study cohorts (*n* = 170,805) revealed that high intake of dietary long-chain ω-3 PUFAs was associated with a reduced risk of UC, while high intake of trans-unsaturated fats may be associated with an increased risk for UC [13].

3. Dietary constituents like maltodextrins and emulsifiers may have a role in the development of inflammatory bowel disease.

#### Grade of recommendation: B, level of evidence: II-3, voting: 81.6% agreement (A: 31.6%, B: 50%, C: 15.8%)

An increasing intake of processed and fast foods has been shown to confer a three- to fourfold greater risk of developing IBD [14]. These foods contain emulsifiers (to improve texture and extend shelf life) and food additives. These emulsifiers or food additives may induce changes in the intestinal barrier and cause microbiome shifts to microbiota with proinflammatory potential, bacterial overgrowth, and impairment of immune responses [15]. Studies have shown a direct effect of commonly used emulsifiers like carboxymethyl cellulose and Polysorbate-80 (P80) on the gut microbiota, in a manner that subsequently drives inflammation in the intestine [15]. Maltodextrin (MDX), an easily digested, branched polysaccharide, is one of the frequently consumed dietary additives. As with other polysaccharide additives, there is evidence of concomitant increase in the incidence of CD in the USA paralleling the greater use of MDX in the American diet [16]. Various studies on animal models have demonstrated a direct effect of maltodextrins on the intestinal mucosal barrier, which translates to exacerbation in intestinal inflammation or increased bacterial burden [17–19].

Dietary interventions including the specific carbohydrate diet (SCD) and the IBD-anti-inflammatory diet (IBD-AID) have shown to promote clinical remission in patients with IBD [20–22]. These dietary interventions exclude prepackaged and processed food products effectively eliminating MDX and other emulsifiers. Although larger clinical studies are needed, the introduction of both SCD and IBD-AID diet has put forward a food for thought on the role of emulsifiers in gut inflammation.

4. Vitamin D may have a protective role in the natural history of inflammatory bowel disease.

#### Grade of recommendation: B, level of evidence: II-2, voting: 83.8% agreement (A: 27%, B: 56.8%, C: 13.5%)

Due to the immune-regulatory functions of vitamin D, links between vitamin D, vitamin D signaling, and IBD have been investigated. Studies on IL-10 knockout mice have shown that concurrent vitamin D receptor (VDR) knockout leads to severe and accelerated IBD, whereas administration of exogenous vitamin D or a VDR agonist in IBD mouse models reduces tumor necrosis factor (TNF)-α and suppresses colitis [23].

Epidemiological studies from different regions of the world have shown that those residing in northern regions, where sunlight exposure and natural synthesis of vitamin D are lower, had a higher incidence of IBD [24–26]. Analysis of the data from the Nurses’ Health Study cohort has shown lower risk for IBD (both CD [HR 0.48, 95% CI 0.30–0.77] and UC [HR 0.62, 95% CI 0.42–0.90]) in women residing in southern regions, when compared to those residing in the north [26]. Among 72,719 women aged 40–73 years who were enrolled in the Nurses’ Health Study and followed up for 1,492,811 person-years, a 25-hydroxyvitamin D (25[OH]D) prediction score was developed and validated against the measured plasma levels of 25(OH)D [27]. A total of 122 new cases of CD and 123 UC were documented. The median predicted 25(OH)D level was 22.3 ng/mL in the lowest and 32.2 ng/mL in the highest quartiles, respectively. Compared with the lowest quartile, multivariate-adjusted HR associated with the highest quartile of vitamin D was 0.54 (95% confidence interval [CI], 0.30–0.99) for CD (*p* [trend] = 0.02) and 0.65 (95% CI 0.34–1.25) for UC (*p* [trend] = 0.17). Compared with women with a predicted 25(OH)D level less than 20 ng/mL, the multivariate-adjusted HR was 0.38 (95% CI 0.15–0.97) for CD and 0.57 (95% CI 0.19–1.70) for UC for women with a predicted 25(OH)D level greater than 30 ng/mL. The study concluded that higher predicted plasma levels of 25(OH)D significantly reduce the risk for incident CD and not so for UC in women. These findings suggest that the effect of vitamin D in the pathogenesis of CD may be stronger than for UC. This is consistent with the fact that 1,25-dihydroxyvitamin D downregulates Th1 cells more than Th2 cells. Nonetheless, a key limitation of the study was the lack of measured vitamin D levels and that it relied more on the predicted level based on dietary and supplement intake, sunlight exposure, race, and body mass index (BMI).

In a prospective nested case–control study using U.S. military personnel data (*n* = 480), vitamin D levels were measured before and after the diagnosis of CD [28]. In this study, no association was found between CD incidence and prediagnosis 25-hydroxyvitamin D levels. Rather, an inverse association was noted between CD and vitamin D levels after diagnosis (OR = 0.74; 95% CI 0.59–0.94; *p* = 0.01). Thus, in contrast to the previous studies evaluating vitamin D levels in IBD, which were retrospective, this prospective study concluded that the vitamin D levels are low after the onset of CD.

5. Breastfeeding may have a protective role in the development of inflammatory bowel disease.

#### Grade of recommendation: A, level of evidence: II-2, voting: 100% agreement (A: 68.4%, B: 31.6%)

Though there were conflicting reports of breastfeeding as protective factor in the development of IBD in the 1980s [29], subsequent data has supported the protective role of the same. A 2004 systematic review with meta-analysis including 17 studies showed that breastfeeding was associated with lower risk of CD (pooled odds ratio 0.67 [95% CI 0.52, 0.86]) and UC (pooled odds ratio 0.77 [95% CI 0.61, 0.96]) [30]. Another systematic review in 2009, which included 7 studies demonstrated significant protective effects of breastfeeding (OR 0.69, 95% CI 0.51–0.94; *p* = 0.02) in developing early onset IBD; however, the protective effect in UC and CD individually was not significant [31]. Following this, many studies from the Western world have reinforced the protective effects of breastfeeding in the development of IBD [32–34]. A study from the Asia-Pacific region consisting of 442 incident IBD cases (186 CD, 256 UC, 374 Asians) from 8 countries in Asia and Australia were compared with 940 controls. On a multivariate model, breastfeeding for > 12 months decreased the odds for both CD (OR 0.10; 95% CI 0.04 to 0.30) and UC (OR 0.16; 0.08 to 0.31) [35]. A recent systematic review, which included 35 studies [36], confirmed that the inverse association between breastfeeding and development of IBD was noticeable in all ethnic populations; however, the magnitude of protection was significantly high among Asians (OR 0.31, 95% CI 0.20–0.48) compared to Caucasians (OR 0.78, 95% CI 0.66–0.93; *p* = 0.0001) in CD. Breastfeeding duration also showed a dose-dependent association, with the strongest decrease in risk when breastfeeding was continued for at least 12 months for CD (OR 0.20, 95% CI 0.08–0.50) and UC (OR 0.21, 95% CI 0.10–0.43) as compared to 3 or 6 months, with a similar risk reduction in both pediatric and adult onset disease.

### Diet as a therapy for IBD

6. Exclusive enteral nutrition (EEN) is as effective as steroids in inducing remission in children with luminal Crohn’s disease.

#### Grade of recommendation: A, level of evidence: I, voting: 97.4% agreement (A: 63.2%, B: 34.2%, C: 2.6%)

Exclusive enteral nutrition (EEN) is defined by the European Society for Clinical Nutrition and Metabolism (ESPEN) to “comprise all forms of nutritional support that imply the use of dietary foods for special medical purposes independent of the route of application.” It is a different form of therapy that possibly targets the pathogenesis, unlike other therapies that target the inflammation and have minimal side effects. The use of EEN as a primary therapy was acknowledged incidentally more than 40 years ago when patients with CD posted for surgery were given EEN. This not only improved the nutritional status, but also disease activity, and significantly reduced surgical intervention.

EEN is primarily classified into three major subtypes: elemental, semi-elemental, and polymeric, depending upon the nitrogen source: amino acids, oligopeptides, or intact protein [37]. The fat and carbohydrate content also vary among these formulae with respect to their complexity and chain length (Table 3). There are several proposed mechanisms for the action of EEN, which include restoration of epithelial barrier, thus preventing bacterial translocation [38], downregulation of proinflammatory cytokines [39], modulation of antigen presentation, changes in gut microbial diversity [40], and reduction in the antigenic load from diet.

**Table 3:** Comparison of types of different exclusive enteral nutrition formulae with respect to protein content

	Elemental	Semi-elemental	Polymeric
Protein	Amino acids	Oligopeptides (hydrolyzed proteins)	Whole protein (casein) or lactoalbumin or whey
Carbohydrate	Glucose polymers	Simple sugars, glucose polymers, or starch	Complex carbohydrates
Fat	Low long-chain triglycerides (LCTs), rich in medium-chain triglycerides (MCTs)	Medium-chain triglycerides	Both MCTs and LCTs
Osmolality (mosm/L)	650–700	375	340

There have been nine meta-analyses that have compared EEN to corticosteroids (CS) in the induction of remission in patients with CD. Of these, three studies included both adults and children [41–43], three included only adults [44–46], and three only children [47–49]. All the three pediatric meta-analyses and subgroup analyses of both adult and pediatric meta-analyses found equal efficacy of EEN and CS in the induction of remission. The latest pediatric meta-analysis reviewed 43 articles, which had evaluated EEN in CD, and of these, 8 finally satisfied the inclusion criteria; randomized and observational studies in children (< 18 years) which compared EEN and CS, with the end-point being remission (the definition of which varied, 8 studies provided information on the proportion of patients in remission) were included [49]. Of these, 5 used polymeric diet; one used elemental diet; one had a separate arm for elemental, semi-elemental, and polymeric diet; and another study did not mention the type of diet. There was no difference in the rates of clinical remission in these children between EEN (*n* = 226) vs. CS (*n* = 225) arm (OR 1.26 [95% CI 0.77–2.05]), as well as among newly diagnosed (*n* = 271; OR 1.61 [95% CI 0.87–2.98]) or relapsed cases (*n* = 133; OR 0.76 [95% CI 0.29–1.98]). Children receiving EEN were more likely to achieve mucosal healing than those on CS (OR 4.50 [95% CI 1.64–12.32]). In the latest Cochrane review, which included only RCTs, EEN had better efficacy than steroids (83% vs. 61% [RR 1.35, 95% CI 0.92 to 1.97]) in children, though the evidence was of low quality. Overall, remission rates on EEN in these studies vary from 60% to 80%. The literature also suggests durability of remission in patients on EEN in the form of reduced CS dependency over 2 years and lower relapse rates over 12 months [50, 51].

There has been little consideration of how factors such as disease location or disease severity influence the clinical outcomes. Although initial data in children suggested better efficacy of EEN in ileal disease [52], subsequent cohort studies [53] and meta-analyses suggested equivalent efficacy regardless of disease location. In a prospective cohort study of 114 children, except for terminal ileal disease (only 4 patients), the remission rates approached ~ 80% in all children irrespective of disease location [53]. A better clinical response to EEN was noted in patients in whom there was an early reduction in the fecal calprotectin levels [54]. EEN has the advantage over CS with respect to improvement in nutritional status [55], reduced incidence of linear growth failure [50], improvement in lean mass [56] and weight, and improvement in biochemical markers of nutritional status [51]. The duration for EEN across studies varies from 6 to 12 weeks, although the usual recommended duration is 6–8 weeks. The route of administration (oral vs. tube feeding) depends on the patients’ tolerability, with no difference in efficacy between the two routes. There is scant data regarding reintroduction of normal diet, and one particular study found no difference in the clinical outcomes between rapid vs. slow reintroduction (over 5 weeks), although most centers follow a gradual reintroduction over a period of 2–3 weeks [57].

Though EEN has been shown to improve extraintestinal manifestations like erythema nodosum in small case series, its role in induction of remission in extraluminal CD is not yet established [58].

7. Exclusive enteral nutrition is effective in adult Crohn’s disease but is inferior to corticosteroids for inducing remission.

#### Grade of recommendation: B, level of evidence: I, voting: 94.8% agreement (A: 55.3%, B: 39.5%)

There are six meta-analyses which have evaluated the role of EEN in adults with CD (Table 3), three included both adults and children [44–46], and three included only adults [41–43]. In these meta-analyses, EEN had lower efficacy than corticosteroids in the induction of remission. In a Cochrane review, which included only randomized controlled trials (RCTs), 45% (87/194) of adult patients on enteral nutrition achieved remission compared to 73% (116/158) of patients on steroids (RR 0.65, 95% CI 0.52 to 0.82). This difference persisted on per-protocol analysis, even after exclusion of patients who had dropped out of the study because of intolerance to EEN (58% vs. 73% [RR 0.82, 95% CI 0.70 to 0.95]). However, recent cohort studies and earlier studies in steroid nonresponsive patients have provided some evidence for efficacy of EEN in adults. In one of the earlier studies, polymeric EEN was compared with elemental diet in patients with steroid nonresponsive CD, and there was ~ 70% improvement in both groups [59]. In a prospective study of 38 patients from China, there was a significant reduction in the Crohn’s Disease Activity Index (CDAI) score and C-reactive protein (CRP) levels after 8 weeks of EEN along with improvement in nutritional status, and reduction in visceral fat area [60]. In another study from New Zealand, 32/38 patients with active CD received EEN for 2 weeks and had significant improvements in disease symptoms (*p* = 0.003), serum CRP (*p* = 0.005), insulin-like growth factor-1 (*p* = 0.006), and fecal calprotectin (FC; *p =* 0.028) [61]. Further treatment with exclusive or partial enteral nutrition maintained initial improvements. In the other prospective study of 65 patients with inflammatory strictures, 74% had symptomatic improvement, 65% had clinical remission, and 54% had radiologic remission on an intention to treat (ITT) analysis. In addition, there was significant improvement in nutritional status and in inflammatory markers [62]. Yet another study of 41 patients with fistulizing/stricturing disease proved the efficacy of EEN with significant reduction in CDAI, 80% rate of clinical remission, 75% rate of fistula closure, and 47% rate of mucosal healing [63]. Further, it has been shown in recent studies that preoperative EEN for patients with complicated CD reduces the need for surgery, decreases the duration of surgery, lowers the rate of need for stoma, reduces postoperative infections and anastomotic leaks, and decreases postoperative recurrence after surgery [43, 64–66]. The rate of adverse events between EEN and CS has been similar (25% vs. 16% [RR 1.39, 95% CI 0.62–3.11]), although the type of adverse events varies between the two groups. Adverse events in the EEN group include heartburn, flatulence, diarrhea, and vomiting, while those of the CS group include acne, moon facies, muscle weakness, and hyperglycemia [43]. Significantly more patients in the EEN group tend to withdraw from therapy as compared to those on steroids (23% vs. 6% [RR 2.95, 95% CI 1.02–8.48]).

Although the meta-analyses have suggested lower efficacy of EEN as compared to CS because of poor quality of trials (high risk of bias), the authors of these meta-analyses suggest larger RCTs to evaluate the efficacy of EEN in adults. Furthermore, because of demonstrated efficacy of EEN in steroid nonresponsive and complicated CD, EEN can be tried in patients who are steroid dependent, refractory, or intolerant, especially when they are nutritionally compromised.

8. There is no difference between elemental and polymeric formulae in terms of efficacy.

#### Grade of recommendation: A, level of evidence: I, voting: 91.9% agreement (A: 51.4%, B: 40.5%, C: 5.4%)

In a Cochrane review, 13 trials compared different formulations of enteral nutrition (based upon nitrogen source), of which four compared elemental to semi-elemental, seven compared elemental to polymeric, and two compared semi-elemental to polymeric [43]. There was no difference in remission rates between any of the protein formulations; specifically, elemental (*n* = 133) and polymeric diet (*n* = 130) had similar efficacy (RR 1.08, 95% CI 0.89–1.31). The review also compared the efficacy of EEN with respect to fat content, and subgroup analysis of seven trials showed similar efficacy between low (*n* = 105, < 20 g fat/1000 kcal) and high fat [*n* = 104, > 20 g fat/1000 kcal) diet containing EEN (RR 1.03, 95% CI 0.85–1.26). Similarly, the diet containing very low fat (< 3 g fat/1000 kcal) also had similar efficacy (RR 1.11, 95% CI 0.84–1.46). The long-chain triglyceride content (< 10% [*n* = 111] vs. > 10% [*n* = 99]) also did not affect efficacy, with a meta-analysis of 6 trials reporting similar remission rates (RR 1.09, 95% CI 0.80–1.31). There was no difference in the rate of adverse events between different formulations (17% vs. 17% [RR 1.01, 95% CI 0.62–1.65]); the withdrawal rates between different formulations were also similar (22% vs. 17% [RR 1.37, 95% CI 0.83–2.25]). However, according to the grade system, the level of evidence for these conclusions was very low.

9. Partial enteral nutrition has been documented to be useful for maintenance of remission in luminal Crohn’s disease along with pharmacotherapy.

#### Grade of recommendation: A, level of evidence: I, voting: 81.6% agreement (A: 31.6%, B: 50%, C: 15.8%)

Though EEN is effective in induction of remission in children and adults (statements 6 and 7), it is usually administered for 8 weeks, following which patients are switched to partial enteral nutrition due to high cost and unpalatability of EN. Partial enteral nutrition (PEN), i.e. supplementation of regular diet with enteral nutrition, is an attractive alternative to immunomodulators and biologics for maintaining remission in patients with luminal CD, as it is devoid of serious adverse effects like infections and malignancy. Since 1987, several studies have suggested that nutritional supplementation with liquid formulae prolongs remission in patients with quiescent CD and improves linear growth in pediatric patients when used before completion of puberty [58, 67, 68]. A systematic review in 2007 assessed the efficacy of enteral nutrition for maintenance of remission in CD [69]. This review included two studies, one of which was a RCT which found significantly lower relapse rates in patients who received half of their total daily calorie requirements as elemental diet and the remaining half as normal diet compared to patients who received unrestricted normal diet (9 of 26 vs. 16 of 25; OR 0.3, 95% CI 0.09 to 0.94) [70]. In the second study, elemental and polymeric feeds (providing between 35% and 50% of patients’ pretrial calorie intake in addition to unrestricted normal food) were found to be equally effective for maintenance of remission and allowing withdrawal of steroid therapy (8 of 19 vs. 6 of 14; OR 0.97, 95% CI 0.24 to 3.92) [71]. A review by Nakahigashi et al. included seven prospective cohort studies (including three RCTs) where patients used enteral nutrition (EN) as a supplement or as a nocturnal tube feeding in addition to their normal food [72]. Four of the six studies among these, which compared the outcomes between the patients treated with and without EN, showed higher rates of clinical remission at 1 year in patients on PEN. A recent systematic review by El-Matary et al. included 12 studies (1169 patients, including 95 children), of which 11 studies showed that EN was either better than or as effective as its comparator in maintaining remission in patients with inactive CD [73]. Only one adult RCT, with low risk of bias, compared EN with a regular diet and found a relapse rate of 34% in the EN group vs. 64% in the control group (*p* < 0.01) after a mean follow up of 11.9 months.

Thus, PEN is more effective than regular diet in maintaining remission for patients with inactive CD. Large, properly designed RCTs of sufficient duration are, however, still required to compare EN with pharmacotherapy.

10. More evidence is required, before elimination diets such as specific carbohydrate diet (SCD), Crohn’s disease exclusion diet (CDED), semi-vegetarian diet (SVD), anti-IBD diet, or low fermentable oligosaccharides, disaccharides, monosaccharides, and polyols (FODMAP) can be recommended as a therapy for Crohn’s disease.

#### Grade of recommendation: A, level of evidence: II-2, voting: 92.5% agreement (A: 65%, B: 27.5%, C: 2.5%)

Though effective for induction of remission in CD, EEN has low compliance, especially in adults [74]. Hence, elimination diets with the exclusion of dietary components hypothesized to affect the microbiome or intestinal permeability have been developed. These include the SCD [20], CDED [75], the BD-AID [21], allergen elimination diet (IgG) [76], SVD [77], the low FODMAP diet [78, 79], and the Mediterranean diet [80].

SCD has been used in IBD with a rationale that malabsorption of disaccharides and complex carbohydrates can cause bacterial overgrowth and intestinal injury. A prospective study which assessed the use of SCD in 10 children with CD found that there were significant clinical and mucosal improvements at 12 and 52 weeks [20]. Resolution of symptoms of IBD has been shown to be associated with an increase in microbial diversity of the fecal samples with SCD [81]. The CDED excludes those dietary components which impair innate immunity, increase intestinal permeability, cause microbial dysbiosis, or allow bacteria to adhere and translocate through the intestinal epithelium in animal models. In a retrospective study of 47 children and young adults with mild to moderate active CD, clinical response and remission at week 12 were obtained in 37 (78.7%) and 33 (70.2%) patients, respectively, and 70% patients had normalization of previously elevated CRP [75] . A recent RCT compared CDED plus PEN with EEN and found that both were equally effective in inducing remission and microbiome changes [82]. A SVD has been used in IBD as vegetarian diet is rich in beneficial bacteria. A 2-year prospective trial by Chiba et al. found that 16/22 patients continued semi-vegetarian diet and 15/16 maintained remission compared with 2/6 in the control group (*p* 0.0003) [77]. The use of a low FODMAP diet can reduce abdominal pain, bloating, wind, and diarrhea experienced by IBD patients [79]. FODMAPs are likely to undergo fermentation and cause increased intestinal permeability and greater risk of tissue injury. However, there is no study to confirm the same. More clinical trials are required to evaluate the efficacy of elimination diets for induction and maintenance of remission in IBD.

11. There is no specific role for exclusive (EEN) or partial enteral nutrition (PEN) for induction or maintenance of remission in patients with ulcerative colitis.

#### Grade of recommendation: B, level of evidence: I, voting: 92.1% agreement (A: 52.6%, B: 39.5%, C: 7.9%)

Enteral nutrition has not been adequately studied in UC. A prospective RCT compared the efficacy of EEN and TPN as an adjunctive therapy to CS in acute severe UC [83]. In both groups, the rates of remission and need for colectomy were similar. No significant change in anthropometric parameters was observed in either group. In spite of this, median increase in serum albumin of 16.7% in the enteral feeding group was significantly greater than 4.6% in the parenteral nutrition group. In another study, 17 patients with severe flare of UC were given enteral feeds with a polymeric formula following a 48-h intensive medical therapy [84]. The formula concentration and volume were increased daily and 14/17 patients tolerated EN well. By day 4, 11 patients attained more than 80% of the caloric requirements and prealbumin levels improved significantly, suggesting a favorable anabolic effect. However, the available data concerning the role of EN in patients with active UC is still inadequate. Additional studies including larger cohorts of patients need to be performed.

### Malnutrition and nutritional assessment in a patient with IBD

12. Patients with inflammatory bowel disease are at a higher risk of malnutrition; hence, all the patients should be screened for it at presentation.

#### Grade of recommendation: B, level of evidence: II-2, voting: 100% agreement (A: 94.4%, B: 5.6%)

There is an increased risk of malnutrition in both adults and children with IBD, especially in patients with CD. In a nationwide inpatient sample between 1998 and 2004, the prevalence of malnutrition was found to be greater in CD and UC patients than in non-IBD patients (6.1% and 7.2% vs. 1.8%, *p* < 0.0001) [85]. The nutritional deficits are more common in patients with active IBD. Malnutrition in patients with IBD may be due to reduced intake, malabsorption, and increased gastrointestinal (GI) losses. In addition to deficiency of various macronutrients, patients with IBD frequently have iron deficiency anemia and deficiencies of other micronutrients like folic acid, magnesium, calcium, zinc, and vitamins A, B_12_, D, E, and K.

Malnourished patients with IBD are more prone to infections [86], which significantly increase the likelihood of hospitalization [87]. In hospitalized patients, malnutrition is an independent risk factor for venous thromboembolism [88], nonelective surgery [89], longer admission [89], and increased mortality [85]. Malnourished children with active IBD have growth failure (in up to 15% and 40%), and this may even precede the diagnosis of IBD by many years [90, 91]. In addition to short stature (which can be seen in up to 30% with active CD) [92], pubertal development is also disrupted due to chronic inflammation and malnutrition. Thus, all pediatric and adult IBD patients must be screened for malnutrition using available screening tools [93, 94]. Children with IBD should also undergo periodic assessment of their nutritional intake, anthropometric measurements, and percentile growth rate.

13. The prevalence of malnutrition in a patient with inflammatory bowel disease depends upon disease subtype, severity, extent, and duration.

#### Grade of recommendation: B, level of evidence: II-2, voting: 100% agreement (A: 76.3%, B: 23.7%)

Malnutrition can occur both in UC and CD but is more frequent in CD as it can affect any part of the GI tract, in contrast to UC, which is restricted to the colon and has few direct malabsorptive effects [95]. The severity of malnutrition in IBD is affected by activity, duration, and extent of the disease, and more importantly, to the extent of inflammatory response which is anorexigenic and drives catabolism. CD patients are at risk even when their disease is quiescent, whereas UC patients generally develop malnutrition only when the disease is active [96]. As mentioned previously, in a nationwide inpatient sample between 1998 and 2004, the prevalence of malnutrition was found to be greater in CD and UC patients than in non-IBD patients (6.1% and 7.2% vs. 1.8%, *p* < 0.0001) [85]. There was increased likelihood of malnutrition among those with fistulizing CD (OR 1.65; 95% CI 1.50–1.82) and those who had undergone bowel resection (OR 1.37; 95% CI 1.27–1.48).

14. Body mass index alone is not sufficient for nutritional assessment of a patient with inflammatory bowel disease.

#### Grade of recommendation: B, level of evidence: II-2 voting: 100% agreement (A: 91.8%, B: 8.2%)

Disease-related malnutrition may not be detected by BMI. Dong et al. conducted a meta-analysis and found that IBD patients had lower BMI than normal controls [97]. Patients with newly diagnosed IBD and those with disease relapse commonly have unintentional weight loss and are underweight. These are more common in CD than UC and are present in approximately 60% and 35% of the new cases, respectively [98]. However, weight alone (and therefore BMI) cannot predict the actual nutritional status of the patient. With the epidemic of obesity and also earlier disease recognition, fewer patients are underweight [99, 100]. Patients with IBD have an alteration of body composition, i.e. reduction in lean body mass with normal or increased fat mass [101]. Sarcopenia is common in IBD patients and such patients have higher rates of both surgical interventions and postoperative complications [102]. Normalization of BMI at 2 years follow up has not been associated with an increase in fat-free mass in CD [103], which supports the fact that BMI changes may not be a good predictor for body composition changes in IBD. Despite all these facts, a significant change in BMI or a very low BMI will need an intervention [104].

15. Dieticians/nutritionists should be involved in nutrition care of patients with inflammatory bowel disease.

#### Grade of recommendation: C, level of evidence: III, voting: 97.4% agreement (A: 89.7%, B: 7.7%)

Management of IBD involves a multidisciplinary approach; however, access to dietetic expertise is generally limited within an IBD multidisciplinary team [74]. Most patients with IBD are unaware of the importance of diet in the management of IBD. In addition to this, common misperceptions can result in dietary changes leading to undernutrition in patients with IBD [105]. Nutritional deficiencies are more common in small bowel CD than colonic CD or UC, and therefore, nutritional care is important in the management of patients with IBD and in the prevention and treatment of malnutrition and micronutrient deficiencies and prevention of osteoporosis in adults and promotion of growth and development in children [27, 106–109]. Inputs of dieticians and specific dietary counseling are therefore as important as prescription of pharmacotherapy and nutritional supplements in patients [110] though the evidence for the same remains poor. Currently, nutritional screening is being done by a dietician with special interest in IBD in many European countries.

16. Dietary recommendations in inflammatory bowel disease: for admitted patients with acute severe ulcerative colitis, adequate oral caloric intake is preferred to bowel rest.

#### Grade of recommendation: A, level of evidence: II-1, voting: 100% agreement (A: 86.8%, B: 13.2%)

Studies comparing bowel rest and oral caloric intake in patients with acute severe UC are lacking. A prospective trial on 36 patients with moderate to severe IBD (UC 27, CD 9) compared those on prednisolone 40 mg/day, intravenous hyperalimentation (IVH) with nil per oral (*n* = 19) with a control group (*n* = 17) who were on normal hospital diet ad libitum. Except for blood replacement and supplementation with albumin in the controls, no intravenous nutrients or vitamins were added [111]. The colectomy rates (47.4% in the IVH group vs. 35.3% in the control group) and mean time to reduction of prednisolone to 10 mg/day (21.2 vs. 23.7 days) were not different between the two groups. IVH with bowel rest was concluded to show no primary therapeutic effect in acute colitis; rather, it had higher rates of complications (up to 15%). In another prospective trial comparing bowel rest with parenteral nutrition (*n* = 27) and oral diet (*n* = 20) in patients with severe, acute, noninfectious colitis receiving 60 mg prednisolone, there were no differences in the operation or mortality rates between the two groups [112]. In the subgroup analysis, medical therapy was successful in 40% (6/15) in the IVH group and in 58.3% (7/12) in controls. However, in a fulminant attack of UC, patients need to be kept nil by mouth, and fluid and electrolyte substitution is required [113] . In malnourished patients, parenteral nutritional support may be indicated [113]. In summary, in acute severe UC, except fulminant colitis, bowel rest with intravenous nutritional support does not provide clinical benefit, and oral caloric intake, if no contraindication exists, is recommended.

17. After stabilization of acute severe ulcerative colitis, a standard diet should be gradually introduced and oral nutritional supplements should not be a routine.

#### Grade of recommendation: B, level of evidence: III, voting: 88.9% agreement (A: 66.7%, B: 22.2%, C: 5.6%)

After stabilization of acute severe UC, if patients have the ability to eat with no other contraindications to oral intake, a standard diet is recommended. Oral nutritional support can be the first step as supportive therapy in patients with inadequate intake. A supplementary intake of up to 600 kcal/day can be added with a gradual increase, without compromising normal dietary intake in adults [114]. In case oral nutritional support is inadequate, tube feeding followed by parenteral nutritional support can be considered [114].

18. For active inflammatory Crohn’s disease, oral diet with high protein is preferred to total parenteral nutrition.

#### Grade of recommendation: A, level of evidence: I, voting: 100% agreement (A: 75.8%, B: 24.2%)

In a prospective, randomized controlled trial on 51 patients with active CD on in-hospital nutritional support for 21 days, patients were randomized to TPN and nil by mouth (*n* = 17), defined formula diet (DFD) administered through a nasogastric tube (*n* = 19), or full palatable meals without restriction on quantity or type plus PPN (*n* = 15) [115]. Clinical remission at discharge was achieved in 71% of patients receiving TPN, 58% on DFD, and 60% on PPN, which were not significantly different. A significant fall in the mean disease activity index (*p* < 0.01) was observed in each group at the end of 21 day therapy, and there was no significant difference in the disease activity index between groups. The remission rates at 1 year in those receiving TPN, DFD, and PPN were 42%, 55%, and 56%, respectively, and these differences were not significant. Thus, the authors concluded that in patients with active CD, bowel rest with TPN did not have additional benefits in the short-term and long-term outcomes. Oral diet is preferred to feeding through a tube, if no clinical contraindication exists. In the active stage of IBD, provision of protein up to 1.2–1.5 g/kg/day is justified considering the activated proteolytic and catabolic response in these patients [114].

19. Once in remission, there is no need for diet modification or restriction and the patients can continue a normal diet as other family members.

#### Grade of recommendation: B, level of evidence: I, voting: 97.3% agreement (A: 73%, B: 24.3%, C: 2.7%)

Resting energy expenditure in IBD has been shown to correlate with disease activity; it decreases with induction of remission [116]. Studies on energy and protein requirement during remission phase of IBD have shown conflicting results. Currently, there is no good evidence that energy and protein requirement in patients with IBD who are in remission is different from that of the general population. A randomized controlled trial conducted in Japan showed half elemental diet is beneficial in maintaining remission in patients with CD [70]. Another study from the UK showed that oral nutritional supplementation with either elemental or polymeric diet in addition to standard therapy improved the chance of steroid-free remission in previously steroid-dependent patients with CD [71]. However, a subsequent Cochrane review concluded that both these studies lacked statistical power to draw a definite conclusion [69]. As mentioned in section 3 (diet as a therapy for IBD), there is no good evidence to support the use of specific diets in IBD. Adhering to a specific diet may put the patient at risk of protein–calorie malnutrition and also result in an unnecessary financial burden.

20. No dietary item in particular is established to cause relapse of disease activity in a patient in remission.

#### Grade of recommendation: B, level of evidence: II-1, voting: 97% agreement (A: 69.7%, B: 27.3%, C: 3%)

A large majority of patients with IBD perceive food as a risk factor and impose dietary restrictions without proper counseling, which results in malnutrition [3, 105, 117–119]. However, there is little evidence from interventional studies to support specific dietary restrictions in IBD [120]. A controlled trial involving 20 patients showed that diet that excluded foods to which a patient was intolerant helped maintain a longer remission in CD compared to unrefined carbohydrate-rich fiber diet [121]. Another RCT concluded that stepwise introduction of food excluding food items that precipitated symptoms is better in maintaining longer remission in CD (where remission was induced with elemental diet alone), compared to tapering steroids and a healthy diet [122]. In patients with UC, a prospective cohort study of 191 patients revealed that high intake of processed and red meat, protein, alcohol, and high sulfur or sulfate increased the likelihood of relapse [123]. Thus, avoidance of specific food items should be based on individual experience, and no generalized recommendation can be made [124].

21. Milk should not be routinely restricted in all patients with inflammatory bowel disease unless patient has severe hypolactasia.

#### Grade of recommendation: A, level of evidence: I, voting: 100% agreement (A: 75.7%, B: 24.3%)

Lactase phlorizin hydrolase (LPH) is the enzyme responsible for hydrolysis of lactose in the brush border of the small intestine. Certain LPH gene polymorphisms have been identified to correlate with lactose sensitivity. Lactose sensitivity has been reported in 70% of patients with IBD in remission irrespective of their LPH gene polymorphism [125]. However, a double blind randomized crossover trial on 30 non-IBD patients who reported symptoms of lactose intolerance revealed that these patients may mistakenly attribute various abdominal symptoms to lactose intolerance. Milk intake of 240 mL or less per day is less likely to produce significant symptoms [126]. A subsequent review also concluded that even though hypolactasia is common in IBD, strict lactose exclusion is usually unnecessary [120].

22. A gluten-free diet is not of a proven value in patients with inflammatory bowel disease.

#### Grade of recommendation: B, level of evidence: II-3, voting: 89.4% agreement (A: 60.5%, B: 28.9%, C: 10.6%)

Self-limitation diets are common in IBD for symptomatic relief. A few case reports and self-reported questionnaire-based studies show significant symptom relief with gluten-free diet (GFD) in the non-celiac IBD patients [127]. The special elimination diets (discussed earlier) which have shown benefit are gluten free [20, 21, 75–80]. A cross-sectional study using a GFD questionnaire in 1647 patients with IBD participating in the Crohn’s Colitis Foundation America (CCFA) Partners longitudinal Internet-based cohort noted that 8.2% patients were on GFD at the time of survey and 19.1% reported previous trial of a GFD [128]. An improvement in symptoms was noted in 65.6% patients and 38% reported fewer or less severe flares. However, no randomized controlled trial or long-term studies on GFD in IBD are currently available. It is also difficult to ascertain whether the symptom improvement is actually due to exclusion of gluten or FODMAP (see below) as many foods high in FODMAPs also contain gluten. More prospective randomized studies are required to assess the role of GFD in IBD.

23. A low FODMAP diet may help in alleviating irritable bowel syndrome -like symptoms associated with IBD.

#### Grade of recommendation: A, level of evidence: I, voting: 96.8% agreement (A: 77.4%, B: 19.4%)

Fermentation of dietary FODMAPs enhances intestinal permeability and thereby results in tissue injury [129]. RCTs and case series have reported that low FODMAP diet reduces irritable bowel syndrome (IBS)-like symptoms in patients with IBD in remission, and improves the quality of life [130, 131]. This has been supported by a recent systemic review and meta-analysis by Zhan et al. that included 2 RCTs and 4 before–after studies (319 patients, 96% in remission). The authors found that a low FODMAP diet is beneficial in reducing GI symptoms like diarrhea (OR 0.24, 95% CI 0.11–0.52, *p* = 0.0003), abdominal bloating (OR 0.10, 95% CI 0.06–0.16, *p* < 0.00001), abdominal pain (OR 0.24, 95% CI 0.16–0.35, *p* < 0.00001), fatigue (OR 0.40, 95% CI 0.24–0.66, *p* = 0.0003), and nausea (OR 0.51, 95% CI 0.31–0.85, *p* = 0.009) in quiescent IBD [132]. However, one has to bear in mind that a low FODMAP diet is restrictive and may compromise a patient who is already malnourished [133]. One also has to balance the symptomatic benefits with potential detrimental effects of the gut microbiome by reducing prebiotics. A low FODMAP diet should be strictly carried out under the guidance of a dietician with gradual reintroduction of FODMAPs according to tolerance after 6 to 8 weeks.

### Nutritional rehabilitation in IBD patients

24. Patients with inflammatory bowel disease should receive adequate calories, proteins, and fats in their diet. The calorie and protein requirement of a patient with IBD in remission is similar to that of a healthy individual. However, the protein requirement is increased in a patient with active disease.

#### Grade of recommendation: B, level of evidence: III, voting: 100% agreement (A: 91.7%, B: 8.3%)

Daily total energy expenditure in patients with CD is similar to other patients [96, 134–137]. In patients with IBD, though the resting metabolic expenditure is increased due to disease activity, the activity energy expenditure decreases due to reduction in physical activity; thus, overall energy requirement may not change [134]. It has been shown that patients with active CD show changes in substrate oxidation similar to those in starvation, but energy expenditure is not altered as in catabolic diseases [136]. Thus, wasting noted in some of these patients is a consequence of malnutrition but not hypermetabolism.

Enteral feeding normalizes these changes rather quickly over a period of a few days. The calculation of calorie requirement of these patients should therefore be based on BMI and the recommended caloric intake for patients with IBD is as follows: BMI < 15, 36–45 kcal/kg/day; BMI 15–19, 31–35 kcal/kg/day; BMI 20–29, 26–30 kcal/kg/day; and BMI > 30, 15–25 kcal/kg/day [135]. A corrected body weight should be used for patients with edema or ascites. Also, an ideal or adjusted body weight (i.e. ideal body weight + [actual body weight − ideal body weight] × [0.25]) rather than actual body weight should be used in undernourished or in obese patients (BMI ≥ 30 kg/m^2^) to avoid under or overfeeding [135].

A protein intake of 0.75 g/kg meets the requirements of most healthy adult population and is the basis for recommended dietary intake of 0.8 g/kg per day in those with IBD [135]. However, protein requirement may be affected by intake of nonprotein calories and underlying nutritional status [135]. Protein requirement in patients with CD is generally increased due to a variety of reasons including increased catabolism, loss of protein due to inflammatory changes in bowel wall, effect of proinflammatory cytokines, and reduced absorption of nutrients. Thus, to achieve a positive nitrogen balance in patients with IBD, maintenance of 1 to 1.5 g/kg protein per day must be provided. Septic or malnourished patients may require proteins up to 2 g/kg/day [96, 135].

25. Patients with inflammatory bowel disease who have anemia should be evaluated appropriately for the cause of anemia and adequately treated.

#### Grade of recommendation: B, level of evidence: III, voting: 100% agreement (A: 85.7%, B: 14.3%)

Anemia is relatively common among patients with IBD due to deficiency of nutrients like iron, folic acid, and vitamin B_12_ and due to GI blood loss and effect of cytokines or due to various pharmacological agents used for treatment. Anemia can affect health-related quality of life, healing of GI lesions in IBD, and inability to do physical activities. A large European study (ECCO-EPICOM study) involving 1871 patients of IBD found a high prevalence of anemia (49% in CD and 39% in UC) [138]. Anemia was attributed to iron deficiency in 12% to 17%, to chronic disease in 10% to 27%, and mixed causes in 23% to 31%. Risk factors for anemia included extensive disease in case of UC and penetrating or colonic disease in case of CD. In a study, from the USA, which included 17,059 adults with IBD, the prevalence of anemia was 32.4% in CD and 27.6% in UC [139]. It was attributed to iron deficiency in 79.2% of those with CD and 85.1% of those with UC. Factors related to anemia in both groups were ≥ 6 IBD-related outpatient visits, female gender, age, and smoking. In another study which included 718 patients with CD and 560 patients with UC, prevalence of anemia at diagnosis was 47% in CD and 33.8% in UC [140]. The risk factors for occurrence of anemia included female gender, elevated CRP in both diseases, and extent and duration of UC and penetrating behavior of CD. Thus, anemia is common in IBD, both at onset of disease and at the time of first diagnosis and also during course of the disease. There is some geographical variation in the etiology of anemia in these patients, but overall iron deficiency remains the most common cause for anemia.

Both oral and parenteral iron have been found to be effective in the treatment of anemia in patients with IBD [141, 142]. Though oral iron has advantages like safety, low cost, convenience, and efficacy, when intestinal absorption is not impaired, it can cause mucosal injury and alteration of microbiota. Impaired uptake in certain situations and issues related to compliance also limit the oral use of iron. On the other hand, parenteral iron has higher efficacy and fast repletion of stores and is safe when certain specific formulations are used. Limitations of parenteral iron therapy include higher cost, risk for iron overload, and anaphylactic reactions. Although bioavailability of oral iron is low, it is generally considered first line therapy for iron deficiency anemia in IBD patients [141, 142]. Novel oral iron preparations like ferric maltol can provide clinically meaningful improvements in hemoglobin without an impact on IBD severity [143]. Such newer oral iron preparations may be used as an alternative to intravenous iron in iron deficiency anemia in IBD patients.

The optimal dose of oral iron in patients with IBD and iron deficiency has not been clearly defined, but a dose of 50–200 mg/day of elemental iron is often recommended [141, 142]. Vitamin C and vitamin D supplementation may improve iron absorption and clinical response to oral iron therapy. Gastrointestinal side effects, including nausea, dyspepsia, diarrhea, abdominal discomfort, vomiting, and constipation, can be seen in up to 20% of patients. Parenteral iron administration is preferred in patients who do not tolerate oral iron or those with severe mucosal disease where response to oral iron is suboptimal. In patients undergoing therapy with TNF inhibitors, concomitant iron supplementation may be prescribed without affecting the disease course or activity [141, 142]. In a network meta-analysis of use of various parenteral iron preparations for treatment of anemia in IBD patients, ferric carboxymaltose was the most effective intravenous formulation, followed by iron sucrose, iron isomaltose, and oral iron [144]. Ferric carboxymaltose was also better tolerated by these patients.

26. Proactive screening for osteopenia and its treatment should be done as per guidelines.

#### Grade of recommendation: A, level of evidence: III, voting: 100% agreement (A: 87.5%, B: 12.5%)

The prevalence of low bone mineral density (BMD) and osteoporosis in IBD patients is high and has been reported to range from 22% to 77% and 17% to 41%, respectively, in different populations [145–149]. The risk factors for low BMD in IBD patients include associated malnutrition or low BMI, vitamin D deficiency, small bowel disease, disease severity, and prolonged use of corticosteroids. Due to low BMD and osteoporosis, the incidence of fractures in persons with IBD is significantly higher than that in the general population [150].

A close monitoring of BMD, better control of disease activity, physical activity, and dietary intake of calcium and vitamin D are recommended to diminish the loss of bone mass in patients with CD and UC [151, 152]. European Crohn's and Colitis Organisation (ECCO) recommends weight-bearing exercise, smoking cessation, and maintaining adequate dietary calcium > 1 g/day to prevent bone loss in patients with IBD [153]. All patients with IBD should be assessed for vitamin D status, and those with low levels should receive adequate vitamin D and calcium supplements to correct the deficiency [154, 155]. Calcium and vitamin D supplementations are also recommended in patients with IBD who are receiving systemic steroid therapy for the duration of therapy and also in patients where *T* score on dual-energy X-ray absorptiometry (DEXA) is less than 1.5 [153].

Among the pharmaceutical options for treatment of osteopenia and osteoporosis, both bisphosphonates (e.g. alendronate, risedronate, ibandronate, and zoledronate) and sodium fluoride have been found to be effective in patients with UC or CD [156, 157]. In a network meta-analysis, zoledronate had the highest probability to be the best treatment to increase lumbar spine bone mineral density in patients with CD, and risedronate showed the greatest power to decrease the risk of adverse effects of drugs [156].

27. Patients should be screened for micronutrient deficiency including calcium, phosphate, magnesium, iron, folic acid, and vitamin B_12_ in an appropriate clinical context.

#### Grade of recommendation: B, level of evidence: III, voting: 100% agreement (A: 75.8%, B: 24.2%)

Patients with IBD are at increased risk of micronutrient deficiency (including folate, iron, magnesium, vitamin B_12_, calcium, phosphate) due to reduced intake of nutrients secondary to anorexia, malabsorption (inflamed mucosa or short bowel after surgical resection), increased losses (fistulae, exudation in gastrointestinal tract), and effect of drugs used in the treatment of IBD [158, 159]. Iron deficiency is the most common cause of anemia in IBD patients [138, 139, 160]. In addition to reduced intake due to anorexia and increased GI losses, impaired iron metabolism contributes to an iron deficient state in IBD. Proinflammatory stimuli, such as lipopolysaccharide, IL-6, and TNF-alpha, cause upregulation of hepcidin that blocks iron from being exported from enterocytes into the bloodstream and causes iron retention in macrophages and monocytes. Traditionally, serum ferritin is used to assess the body iron store and for diagnosis of iron deficiency. However, interpretation of serum ferritin levels in patients with IBD needs caution as ferritin is an acute phase reactant. In patients without clinical symptoms of active IBD and normal C-reactive protein level, a serum ferritin level of < 30 mcg/L can be considered suggestive of iron-deficiency state. However, in the presence of inflammation, the lower limit of this parameter consistent with normal iron stores may be up to 100 mcg/L. Low iron values and < 16% transferrin saturation can also be taken as supportive evidence of iron deficiency. Deficiency of vitamin B_12_ and folic acid can result in macrocytic anemia. As B_12_ is absorbed in the terminal ileum, patients with CD in this segment are more prone to B_12_ deficiency, as compared to UC in which the disease is usually limited to colon [161]. In a retrospective case–control study by Yakut et al., 10/45 (22%) patients with CD had vitamin B_12_ deficiency, which was significantly higher than UC patients (4/93, 4.3%, *p* = 0.014) and controls (4/53, 7.5%) (*p* = 0.039). [161]. Risk of vitamin B_12_ deficiency is highest in those with ileal or ileocecal resection. UC patients who have undergone proctocolectomy with ileoanal pouch anastomosis also have vitamin B_12_ deficiency [158]. Diagnosis of vitamin B_12_ deficiency is made by documenting its low level in blood (< 200 pg/mL or < 150 pmol/L). However, if suspicion of vitamin B_12_ deficiency is high and blood level is found to be normal, measurement of methylmalonic acid and homocysteine levels in the blood can be considered as these appear to be more sensitive than measurement of serum vitamin B_12_ level [158]. Patients with CD who have terminal ileal resections of > 60 cm need lifelong B_12_ replacement [162].

As folic acid is not stored in the body, reduced intake or increased requirements rapidly result in a folic acid-deficient state. In addition, sulfasalazine, azathioprine, and methotrexate can cause folate deficiency, by inhibition of dihydrofolate reductase and cellular uptake of folate [158]. In the study by Yakut et al., abnormal serum folate levels (< 3 ng/mL) were found in 28.8% of the CD patients, 8.8% of UC patients, and 3% of controls [161]. Other studies have also reported similar results [163, 164]. Folate deficiency is assessed by levels of serum and red blood cell folate. If these tests are negative and suspicion for folate deficiency is high, homocysteine levels can also be assessed. This is potentially more sensitive, although less specific, as hyperhomocysteinemia also occurs with deficiencies of vitamin B_6_ and vitamin B_12_ [158]. Folate supplementation of 1 mg/day is usually sufficient to replenish deficient folate stores within 2–3 weeks. Routine folate supplementation may be considered in pregnant females with IBD and those receiving methotrexate or sulfasalazine.

Magnesium deficiency has been reported in 13% to 88% of IBD patients in various studies [165–167]. Magnesium is an important cofactor in a large number of enzymatic processes. Most of the body magnesium is in the bones in the form of hydroxyapatite crystals and only < 1% of magnesium stores are present in the blood [158]. Magnesium deficiency in IBD is caused by increased GI losses. This results in reduced osteoblastic activity and, thus, hampers the mineralization of bones. Magnesium deficiency can also impair functioning of parathyroid gland and secondarily lead to hypocalcemia. Patients may present with abdominal cramps, impaired healing, fatigue, and bone pain. The diagnosis of magnesium deficiency can be made by measuring serum levels or its 24 h urinary excretion (more accurate) [158]. Magnesium deficiency can be treated by supplementation, but most of the magnesium salts are known to cause diarrhea. Magnesium heptogluconate or magnesium pyroglutamate may be better tolerated, especially if mixed with oral rehydration solution or beverages [158].

Zinc deficiency is relatively common in patients with chronic diarrhea, malnutrition, and various catabolic states [163, 168]. Thus, IBD patients should be considered for supplementation even in the absence of overt signs or symptoms of zinc deficiency. In addition to the micronutrients mentioned above, IBD patients can develop deficiency of many other micronutrients like chromium, selenium, copper, etc. Micronutrients, particularly vitamin A, vitamin C, and zinc, play an important role in wound healing and also enhance the immune response, which is an important aspect in IBD patients [169].

The deficiency of multiple micronutrients may occur simultaneously; thus, IBD patients who develop overt deficiency of one micronutrient may be considered for supplementation of various micronutrients empirically. However, plasma concentrations of several trace elements and vitamins may decrease in patients with IBD because of the systemic inflammatory response [170]. Thus, low values may not necessarily indicate deficiency and a reliable clinical interpretation for plasma zinc can be made if CRP is <20 mg/L, Similarly, for reliable interpretation of plasma selenium and vitamins A and D, CRP should be <10 mg/L and for vitamins B_-6_ and C, the CRP should be <5 mg/L.

28. Except for patients with stricturing Crohn’s disease, there is no evidence for recommending either a low or a high fiber diet for patients with inflammatory bowel disease.

#### Grade of recommendation: A, level of evidence: I, voting: 100% agreement (A: 87.1%, B: 12.9%)

Patients with IBD report several dietary issues both in the active phase and during remission [171]. One RCT conducted in the UK on 352 CD patients showed that there was no significant difference in clinical outcomes detected among patients with low or high fiber diet on CD activity [172]. This finding was confirmed in a systematic review of 23 RCTs (10 on UC, 12 on CD, 1 on pouchitis, including 1296 patients in remission or active disease). The studies recruited patients with varying severity of the disease (remission, active, and mixed), used a variety of supplements (germinated barley, inulin, oligosaccharide/inulin mix, and psyllium) or dietary advice (high fiber and low fiber) over a range of differing time periods (2 weeks to 29 months), and recorded a variety of clinical outcomes (remission rates, remission duration, response rates, and disease activity) using varying indices in view of which a meta-analysis was not possible. The review found no evidence that fiber should be restricted in IBD patients except during flares [173]. In patients with IBD without overt risk of obstruction, the restriction of dietary fiber is unnecessary, but all patients should be appropriately monitored for their tolerance to fiber intake.

A cohort study was conducted by collecting a completed 26-item dietary survey from 1619 participants in the Crohn’s and Colitis Foundation of America Partners Internet cohort [174]. Eligible individuals were in remission as determined by the disease activity index at baseline and completed a follow up survey 6 months later. Among CD participants, those in the highest quartile of fiber were significantly less likely to have a flare, crude OR 0.57 (95% CI 0.38–0.86). In UC patients, high fiber consumption was not associated with likelihood of flare, with crude and adjusted ORs for quartile 4 vs. 1 of 1.38 (95% CI 0.74–2.60) and 1.82 (95% CI 0.92–3.60), respectively [174]. In summary, there is little evidence that fiber should be restricted in IBD patients’ diets, except during an active flare, as low fiber diets may increase colonic microbiota dysbiosis.

29. Patients with inflammatory bowel disease should refrain from alcohol as it may worsen the symptoms of the disease.

#### Grade of recommendation: B, level of evidence: II-3, voting: 93.5% agreement (A: 64.5%, B: 29%, C: 3.2%)

Alcohol is one of the socioenvironmental factors which have been recognized to cause a flare of symptoms in IBD patients [175]. In addition to its proinflammatory effects, alcohol disrupts the absorption of multiple micronutrients and vitamins, resulting in fatigue and reduced bone mineral density [175]. In a prospective cohort study, 191 UC patients in remission were followed for 1 year and 52% relapsed [123]. Alcohol (OR 2.71 [95% CI 1.1–6.67]) in the top tertile of intake increased the likelihood of relapse as compared with the bottom tertile [123]. Another prospective cohort study recruited 21 patients: 8 with inactive UC, 6 with inactive CD, and 7 healthy controls. The study showed that 1 week of moderate consumption of red wine in inactive IBD was associated with a significant increase in intestinal permeability (*p* = 0.028), thus suggesting that patients with inactive IBD who drink red wine daily may be at an increased long-term risk for disease relapse [176]. In a cross-sectional study, which recruited 129 patients (52 CD, 38 UC, and 39 IBS), 75% of IBD (*n* = 42) and 43% of IBS (*n* = 9) patients reported a worsening of GI symptoms after alcohol consumption (*p* = 0.01) [177].

The largest case–control study from China, including 1308 UC patients and 1308 controls, concluded that UC was, compared to abstainers or rare users of alcohol, associated with previous light and heavy alcohol consumption (OR [95% CI] 1.26 [1.07–1.49] and 1.45 [1.12–1.88], respectively) [178]. However, these associations were not seen in the multivariate analysis. In a nested case–control study from a prospective cohort of healthy European individuals at enrolment, the role of different facets of alcohol consumption (lifetime, at the time of enrolment into the study) for the risk of IBD (after adjustment of the smoking status) was assessed. The authors found no association between alcohol consumption and the risk of IBD [179]. Thus, though the Asian data suggests that alcohol maybe a risk factor for IBD, Western data provides conflicting results. The increase in IBD in Asia has been attributed to westernization of the culture, of which alcohol is just one marker and therefore not a primary risk factor for development of IBD [180, 181].

However, as alcohol is well known to be both proinflammatory and directly harmful to gut barrier function [182], patients with IBD should refrain from consuming moderate to heavy amount of alcohol.

30. Patients of inflammatory bowel disease should be encouraged to refrain from smoking.

#### Grade of recommendation: A, level of evidence: I, voting: 100% agreement (A: 88.2%, B: 11.8%)

The effect of smoking on IBD has been established over the decades; however, the exact mechanism of how smoking affects IBD remains an area of research [175]. The mechanisms by which smoking exerts its impact on disease and the rationale for the dichotomous effect in patients with CD and UC are not clear. Recent evidence suggests that smoking induces alterations in both the innate and adaptive immune systems [183].

A multicenter prospective cohort study included 573 CD patients in clinical remission with varying smoking habits [184]. A total of 148 continuing smokers, 190 non-smokers, 160 former smokers, and 75 quitters were included. In comparison with nonsmokers, continuing smokers relapsed more frequently with an incidence rate ratio of 1.53 (95% CI 1.10–2.17). Former smokers and quitters had similar relapse incidences compared with non-smokers. Smoking was an independent predictor for disease relapse in the multivariate analysis (hazard ratio 1.58 [95% CI 1.20–2.09]). In a retrospective analysis of 426 patients with CD from India, 59 were ever-smokers, and smoking had no effect on disease location, behavior, age at onset, perianal disease, extraintestinal manifestations, or medical and surgical treatment requirements [185]. However, current smoking status was found to be associated with a greater use of immunosuppressants (adjusted odds ratio [95% CI] 4.4 [1.1–18.1]) in CD cases in China [186].

A systematic review and meta-analysis showed that current smokers with CD were at increased risk of intestinal resection compared to never smokers (HR 1.27, 95% CI 1.08 to 1.49); however, there was no difference in the need for surgery when former and never smokers were compared (HR 1.11, 95% CI 0.95 to 1.30) [187]. Another meta-analysis of 33 studies revealed that as compared to non-smokers, smokers had increased odds of flare of disease activity (OR 1.56; 95% CI 1.21–2.01), flare after surgery (OR 1.97; 95% CI 1.36–2.85), need for first surgery (OR 1.68; 95% CI 1.33–2.12), and need for second surgery (OR 2.17; 95% CI 1.63–2.89), and quitting smoking was found to ameliorate this [188]. Smoking makes surgical complications more common after colorectal surgery for any indication [189, 190]. Passive smoking and light smoking (< 10 per day) are as bad as heavy smoking [191, 192]. The adverse effects of smoking are more pronounced in women than in men with Crohn’s disease [193]. In patients with UC, there was no difference in the need for colectomy when current smokers were compared with never smokers (HR 0.98, 95% CI 0.67 to 1.44). Former smokers with UC were at increased risk of colectomy (HR 1.38, 95% CI 1.04 to 1.83) compared to never smokers.

Despite the lack of clear benefit in IBD, advice for smoking cessation should still be incorporated into guideline on the management of IBD given the health benefits of smoking cessation. Smokers should be offered a referral to a smoking cessation service where they should be offered behavioral therapy (e.g. cognitive behavioral therapy), in combination with pharmacotherapy (nicotine replacement, bupropion or varenicline). Bupropion and varenicline should not be given to those under 18, and pregnant women should be advised about the risks and benefits of nicotine replacement therapy. Without support, there is a less than 10% likelihood of long-term abstinence in smokers attempting to stop, but these interventions increase success rates substantially [194].

31. There is no scientific evidence to recommend probiotics as a food supplement.

#### Grade of recommendation: A, level of evidence: I, voting: 87.1% agreement (A: 67.7%, B: 19.4%, C: 9.7%)

Altered gut bacteria and bacterial metabolic pathways are two important factors in initiation and progression of IBD [195]. However, efficacy of probiotics in remission of patients with IBD has not been characterized. A systematic review and meta-analysis to examine the efficacy of probiotics in IBD (22 RCTs) showed no benefit of probiotics over placebo in inducing remission in active UC (relative risk [RR] of failure to achieve remission = 0.86; 95% CI = 0.68–1.08) [196]. However, when only trials of VSL#3 (probiotic mixture containing eight bacterial strains, the De Simone formulation) were considered, there appeared to be a benefit (RR = 0.74; 95% CI = 0.63–0.87). This finding was confirmed by another systematic review and meta-analysis (nine trials) on efficacy of probiotics in patients with CD, in CD after surgery, in children, and in active CD [195]. The results showed that probiotics had no significant effects on CD (95% CI 0.7–1.0, *p* = 0.07, RR = 0.87). Probiotics have also been tried both for primary and secondary prevention of pouchitis. In a trial from Italy, 40 patients with pouchitis in clinical and endoscopic remission were randomized to receive VSL#3 and placebo and followed up for 9 months. Three patients (15%) in the VSL#3 group and 20 (100%) in the placebo group relapsed (*p* < 0.001) [197]. VSL#3 has also been tried for secondary prevention in patients with recurrent or refractory proctitis [198]. In a British study, 17/20 (85%) patients on VSL#3 maintained remission, as compared to 1/16 (6%) on placebo (*p* < 0.0001), thus resulting in better quality of life [198]. The overall quality of these studies was low. Probiotics can be considered for prevention of relapsing pouchitis, but there is insufficient evidence to recommend primary prophylactic probiotics after pouch surgery, or in patients at higher risk of pouchitis, such as those with preoperative extraintestinal manifestations, primary sclerosing cholangitis, or high titers of p-ANCA.

32. The nutritional status of patients with inflammatory bowel disease should be optimized prior to elective surgery for a better outcome.

#### Grade of recommendation: B, level of evidence: III, voting: 100% agreement (A: 93.6%, B: 6.4%)

A proportion of patients with IBD (up to 85%) awaiting surgery have malnutrition, which adversely affects the surgical outcome [85, 95, 199]. Thus, assessment of nutritional status in the preoperative setting is essential, and such screening should include nutritional risk score assessment, details of oral intake, and degree of weight loss. There are validated screening tools, such as the Nutritional Risk Score (NRS) [200], Malnutrition Universal Screening Tool (MUST) [94], and Subjective Global Assessment [201], and these may be used in adults. In children, pediatric modification of these scores may be used, but this is an unclear area [93]. The ESPEN guidelines on nutrition in surgery suggest that severe malnutrition can be assessed by (a) weight loss > 10 to 15% within 6 months, (b) BMI < 18.5 kg/m^2^, and (c) Subjective Global Assessment Grade C or Nutritional risk score 5 (both suggest severe malnutrition) [202]. Recently albumin level of less than 3.0 g/dL has been added to this [203, 204]. BMI alone may not be sufficient to assess malnutrition in overweight patients as their BMI will remain in normal range even after weight loss.

Preoperative nutritional support can improve surgical outcomes. A review of preoperative nutritional conditioning in patients with CD (14 original studies [4 prospective, 1 RCT] and 15 reviews) confirmed that malnutrition was a major factor for postoperative complications and suggested that both enteral and parenteral nutrition routes were efficient in reducing postoperative morbidity [205]. This study also suggested that the nutritional guidelines for non-IBD surgery can be applied to IBD [205].

33. If the nutritional goals cannot be met with an oral diet alone, oral nutritional supplements (ONS) or enteral nutrition should be initiated prior to surgery/perioperative phase.

#### Grade of recommendation: B, level of evidence: III, voting: 100% agreement (A: 84.4%, B: 15.6%)

As mentioned above, if the nutritional screening suggests malnutrition, it needs to be corrected prior to surgery. If the dietary intake is not sufficient, there is clearly a need for artificial nutrition. The decision on route of nutrition depends on the ability of the patient to eat; absorptive capacity of the intestines may be affected in patients with extensive disease, those with previous resection, presence and location of fistula/ae, and active infection. In patients who can eat and have a nutritional risk score > 3, oral nutritional support for 7 days and enhanced recovery protocol may be adequate [205]. Oral nutritional support can provide 600 kcal without compromising normal oral intake in adults [114]. In high-risk patients (as defined above), if > 60% of energy needs can be provided by EN, it should be provided for 6 weeks before surgery. If > 60% of nutritional requirement cannot be provided by EN, parenteral nutrition will be needed as a supplementary to EN or exclusively if the GI cannot be used preoperatively and/or postoperatively [114]. In the postoperative setting, if oral intake is not possible for 5 days or more than 50% of required intake is not possible for 7 days, nutritional therapy should be initiated [202]. Whenever possible, oral intake or EN should be initiated within 24 h after surgery [206, 207]. In patients who have hypoalbuminemia, though feeding is an important supportive measure, nutritional support alone is very unlikely to restore low albumin levels. The evidence to support the use of intravenous albumin is poor and underlying sepsis and inflammation should be adequately controlled [208].

34. In elective surgery, the early/enhanced recovery after surgery protocol should be followed in the perioperative period

#### Grade of recommendation: C, level of evidence: III, voting: 100% agreement (A: 72.4%, B: 27.6%)

Early/enhanced recovery after surgery **(**ERAS) aims at accelerated recovery with reduction in hospital stay. It includes prehospitalization phase (patient and family education, pain management plan, patient optimization), preoperative phase (limit fasting and allow light meals up to 6 h prior to surgery, and pre-medications), operative phase (analgesia with opioids, adequate fluids, prophylaxis for nausea and vomiting, normoglycemia, avoid tubes and drains), postoperative phase (early nutrition, early mobilization, analgesia, nausea/vomiting management, avoiding overinfusion of IV fluids and prophylaxis against thromboembolism), and postdischarge phase (continued care and follow up). The ERAS protocol is associated with early recovery of bowel function, reduction in hospital stay, and reduction in pain medication and cost [209, 210]. This can be extrapolated in IBD patients.

### Special situations: surgery, ostomies, pregnancy, lactation

35. Oral diet/enteral nutrition should be started as soon as the patient can tolerate in the postoperative period

#### Grade of recommendation: A, level of evidence: I, voting: 100% agreement (A: 79.4%, B: 20.6%)

The recommended perioperative management of patients with IBD undergoing surgery accords with general ESPEN guidelines as for abdominal surgery [114]. The role of early postoperative EN after any GI surgery has often been controversial with opposing results. A few trials with early EN have shown lower incidence of septic complications and faster wound healing while others have not. A Cochrane review in 2006 (13 RCTs, 1173 patients) concluded that early commencement of postoperative EN compared to traditional management (no nutritional supply) was associated with fewer complications (occurrence of wound infections and intra-abdominal abscesses and postoperative complications such as acute myocardial infarction, postoperative thrombosis or pneumonia, anastomotic leakages, mortality, length of hospital stay, and significant adverse effects) in patients undergoing GI surgery [207]. Another meta-analysis included 15 RCTs (1240 patients) and evaluated surgical outcomes following nutritional provision proximal to the anastomosis within 24 h of GI surgery compared with traditional postoperative management [211]. Patients with early postoperative feeding showed a statistically significant reduction in total postoperative complications (45%) (OR 0.55; 95% CI 0.35–0.87, *p* < 0.01). Early feeding did not affect anastomotic dehiscence (OR 0.75; 95% CI 0.39–1.4, *p* = 0.39), mortality (OR 0.71; 95% CI 0.32–1.56, *p* = 0.39), days to passage of flatus (weighted mean difference [WMD] − 0.42; 95% CI −1.12 to 0.28, *p* = 0.23), first bowel motion (WMD − 0.28; 95% CI − 1.20 to 0.64, *p* = 0.55), and reduced length of stay (WMD − 1.28; 95% CI − 2.94 to 0.38, *p* = 0.13). Thus, early EN should be considered in IBD patients postoperatively as it offers significant benefits with regard to postoperative recovery and infection rate.

36. In the postoperative period, if oral diet cannot be resumed within 7 days then enteral/ parenteral nutrition should be initiated.

#### Grade of recommendation: A, level of evidence: I, voting: 97.1% agreement (A: 70.6%, B: 26.5%, C: 2.9%)

Nutritional support in IBD patients undergoing surgery is indicated in patients with malnutrition, those who are unable to eat for more than 7 days perioperatively, even without significant malnutrition, and in those who cannot maintain oral intake above 60% to75% of recommended intake for more than 10 days. In all the aforementioned situations, nutritional support (preferably by the enteral route) should be initiated without delay. Inadequate oral intake for > 14 days is associated with a higher mortality. In a systematic review and meta-analysis of 29 RCTs (2552 patients), EN was beneficial for reduction of any complication (RR 0.85; 95% CI 0.74–0.99; *p* = 0.04), infectious complication (RR 0.69; 95% CI 0.56–0.86; *p* = 0.001), anastomotic leak (RR 0.67; 95% CI 0.47–0.95; *p* = 0.03), intra-abdominal abscess (RR 0.63; 95% CI 0.41–0.95; *p* = 0.03), and duration of hospital stay (WMD− 0.81; 95% CI − 1.25 to 0.38; *p* = 0.02) [212]. Thus, in the postoperative period, oral feeds should be initiated at the earliest, i.e. within 7 days. If this is not feasible, parenteral nutrition should be considered.

37. In Crohn’s disease patients with a fistula, the type of diet depends upon the location of fistula–oral feeds for distal (low ileal or colonic) and low output fistula, and partial or exclusive parenteral nutrition for proximal and high output fistula.

#### Grade of recommendation: B, level of evidence: II-3, voting: 100% agreement (A: 51.9%, B: 48.1%)

There is no RCT which will guide the best practices in dietary management of fistulizing CD. Early data on the role of TPN in IBD or enterocutaneous fistulae (ECF) came from small, retrospective studies that included patients with different etiologies, and the nutritional support was given for only short periods so no definite conclusions can be drawn from these. It is logical to assume that high-output intestinal fistulae need parenteral nutrition. Other indications for TPN include intolerance to EN, inability to meet nutritional needs by enteral feeding alone, and anastomotic leaks after surgery [213–215]. Poor nutritional status at the time of presentation is an important determinant of increased postoperative morbidity in patients undergoing surgery for ECF of any etiology [216]. An early report on 132 patients with ECF of whom 48 had it due to CD demonstrated that the use of TPN led to weight gain and metabolic restitution [217]. In a retrospective study on patients with CD refractory to conventional medical management, TPN improved disease activity and nutritional status and led to healing of the fistulae in 63% patients [218]. EN is preferred over TPN because of higher risk of complications associated with the latter [219]. Evidence for the usefulness of enteral nutrition EN too is based on case series. In a study by Yan et. al., low output ECF (defined as draining < 200 mL/24 h) closed in 30/48 (63%) after 3 months of EN therapy with a short-peptide-based EN provided by continuous infusion through a nasogastric tube [220]. In another small prospective study from China, 75% of the ECF due to CD healed on 12 weeks of EEN [63]. Fistula closure was accompanied by significant clinical remission and mucosal healing, along with improvements in the nutritional status and markers of inflammation [63, 220]. In a retrospective analysis, patients with CD and ECF had a significantly lower risk of postoperative intra-abdominal septic complications if they had received preoperative EEN [221].

38. Inflammatory bowel disease patients with pregnancy should be specifically evaluated for iron and folate deficiency and replacement done accordingly. Recommended Dietary Allowances (RDA) for pregnancy and lactation should be followed.

#### Grade of recommendation: B, level of evidence: III, voting: 100% agreement (A: 77.3%, B: 22.7%)

Anemia occurs in 25% to 52% of pregnant women with IBD in Asia and deficiency of iron and folic acid usually results from increased metabolic demand [221]. Iron deficiency anemia is reported to occur in 36% to 76% of patients with IBD [222]. Folate deficiency occurs less commonly in IBD but is more prevalent in CD (22.2% to 28.8%) than in UC (4.3% to 8.8%). It can result from extensive small bowel disease or resection and inhibition of folate absorption by sulfasalazine or associated hemolysis. Thus, all patients with IBD who are pregnant need to be screened for iron and folate deficiencies as per standard guidelines, and when detected, appropriate treatment needs to be initiated. Those who are not deficient should receive supplementations as per the recommended dietary allowances for pregnancy and lactation [223, 224]. However, it should be noted that there is no conclusive evidence that routine prenatal supplementation of iron and folic acid improves maternal or infant clinical health outcomes even though appropriate supplementation may improve maternal hematologic indices [225, 226].
